# A relational approach to co-create Advance Care Planning with and for people living with dementia: a narrative study

**DOI:** 10.1186/s12904-024-01632-y

**Published:** 2025-01-08

**Authors:** Tharin Phenwan, Judith Sixsmith, Linda McSwiggan, Deans Buchanan

**Affiliations:** 1 School of Health Sciences, University of Dundee, Dundee, UK; 2 School of Medicine, University of Dundee, Dundee, UK; 3 NHS Tayside and Dundee Health and Social Care Partnership, Dundee, UK

**Keywords:** Dementia, Narrative approach, Narrative analysis, Advance Care Planning, Palliative care, Online method, Relationality, Interdependence, Qualitative research

## Abstract

**Background:**

Discussing Advance Care Planning (ACP) with people living with dementia (PwD) is challenging due to topic sensitivity, fluctuating mental capacity and symptom of forgetfulness. Given communication difficulties, the preferences and expectations expressed in any ACP may reflect family and healthcare professional perspectives rather than the PwD. Starting discussions early in the disease trajectory may avoid this, but many PwD may not be ready at this point for such discussions. Consequently, the optimal timing to discuss an ACP with and for PwD is undetermined. This study explored the changing needs of PwD and experiences of social contexts that influence ACP initiation and revision and aimed to identify the optimal time to discuss an ACP with PwD.

**Methods:**

Narrative online and telephone interviews were conducted with 13 PwD and 23 family carers. Participants were recruited via the Join Dementia Research (JDR) Platform. Narrative analysis was used to identify patterns in the data, generating three narratives: Shifting Expectations; Relational Interdependency and Trigger Points.

**Results:**

The Shifting Expectations narrative indicated that PwD’s needs shifted to co-constructed needs with their family as PwD’s independence declined. This was reflected in the Relational interdependency narrative where PwD almost always co-created and revised their ACPs with trusted key persons who provided relational support. The Trigger points narrative indicated various points in time when PwD can effectively initiate and revise their ACPs, ranging from before the diagnosis to years afterwards, challenging the current suggestion of an early ACP initiation.

**Conclusions:**

This study highlighted the changing co-constructed needs between PwD and their families that influence how PwD initiate and revise their ACP. The identification of ACP trigger points - the pivotal events throughout the dementia journey - that prompt PwD and family members to discuss their ACPs were suggested, indicating that PwD can initiate and revise their ACPs throughout the disease trajectory provided relational support is available whereby key persons involved in their care are involved and agree with the decisions being made. Therefore, an alternative, relational approach to ACP with and for PwD is recommended.

**Supplementary Information:**

The online version contains supplementary material available at 10.1186/s12904-024-01632-y.

## Introduction

Dementia is an umbrella term used to describe various neurodegenerative conditions that affect a person’s memory, cognitive abilities and capacity to make decisions [1]. Currently, an estimated 50 million people worldwide are living with dementia; this number is expected to rise to approximately 152 million by 2050 due to increasing life expectancy [2]. In the UK, more than 1 million people are projected to have dementia by 2025, with the number expected to double by 2050 [3].

Given that dementia is not a curable disease, dementia policies aim to support people living with dementia (PwD) to live independently in the community for as long as possible, with support from families, friends, healthcare professionals (HCPs) or charities and assistance from technology [4]. The dementia policies are paralleled with strategies targeting HCPs to ensure that PwD will receive good quality care and to minimise the escalating cost of dementia care [5].

Good quality care is often associated with person-centredness and is encouraged since the person’s inputs in relation to their care are being heard thus facilitating control over their lives in care [6]. One strategy to safeguard PwD’s personal values, life goals and preferences of future care is through Advance Care Planning (ACP) [7].

ACP is an iterative process which “…s*upports adults at any age or stage of health in understanding and sharing their personal values*, *life goals*, *and preferences regarding future medical care”* [7](p. 286). The advantages of creating an ACP for PwD include improved quality of life [8], a decrease in non-beneficial medical care towards the end-of-life [9] and consistent care goals for the individuals that would support their personal values and care priorities [10] as well as the reduced likelihood of unnecessary hospitalisations [11].

Nevertheless, it is challenging to initiate and revise an ACP with and for PwD due to the potential sensitivity of the topic, their fluctuating mental capacity [8] and symptoms of forgetfulness and anxiety which may affect their capability to express their preferences [12]. Further problems may occur as the disease progresses when family members, family carers (all hereafter called ‘carers’) and HCPs become more involved in the ACP process (including revising and actioning the ACP) yet their input might not reflect PwD’s own preferences [13]. There is also little information regarding the optimal timing to initiate and revise and ACP with and for PwD. The literature suggests that an early initiation of ACP with PwD during the ‘window of opportunity’ period is preferred [14–16]. However, this window of opportunity has not been explicitly identified throughout the dementia trajectory thus requires further investigation.

## Methods

### Study aim, approach and design

The study aimed to: (i) explore the changing needs of PwD as the disease progresses and examine how that affects their ACPs; (ii) examine the social contexts around PwD that influence the ACP process and (iii) identify the optimal time to initiate and revise an ACP. The research questions were:

- How do PwD’s daily lives change over time and how do these changes affect their expectations for the future?
- How does the social context of PwD affect the initiation and revision of their ACPs?
- Are there optimal times for initiating and revising ACPs with and for PwD?

To provide a guiding framework for the study, Bronfenbrenner’s bioecological theory was adapted as the study’s theoretical framework [17, 18]. Bronfenbrenner posits that individuals (in this instance, PwD) are nested under several environment systems. This includes their immediate setting (family, friends) to the more remote and abstract environment of cultural influences, societal values and policies which will influence each other over time [19].

**Fig. 1:**
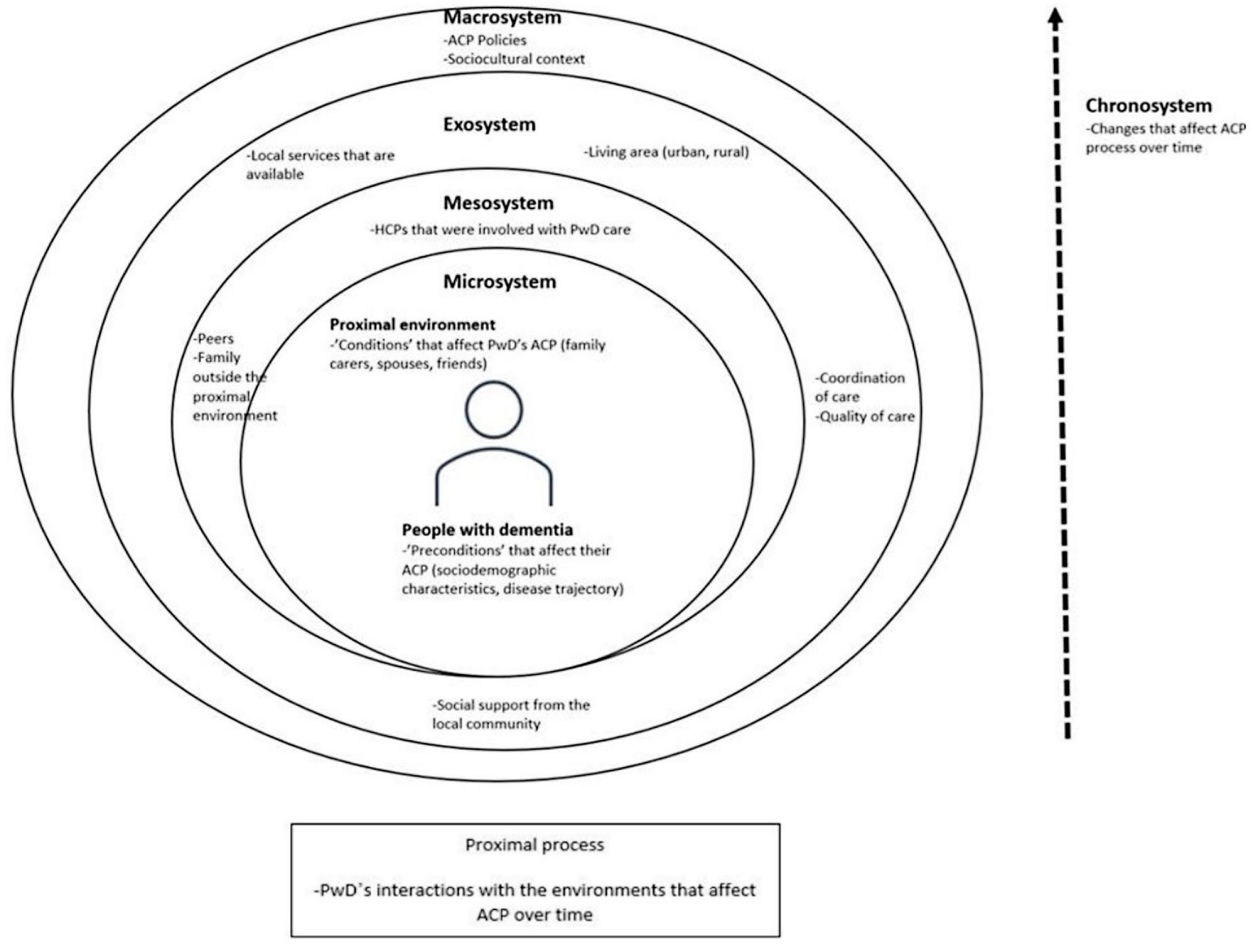
Theoretical framework

The microsystem (PwD and their interactions with carers and friends), mesosystem (the influences from HCPs and extended families) and exosystem (coordination of care and quality of care that PwD received) of Bronfenbrenner’s bioecological theory and how each system influenced the ACP process over time (chronosystem) were examined. Another study was also undertaken to explore the macro-influence of dementia policies over the ACP process for PwD (see Fig. 1).

The study adopted a social constructionist approach which acknowledges the existence of multiple realities [20, 21], the integral importance of the social world around us in shaping our understandings and actions, and the involvement of the researcher in co-creating knowledge. To explore constructions around ACPs for PWD, Stephen and Breheny’s integrated narrative approach was employed [22]. In line with social constructionism, this narrative approach combines three levels of narratives:

- Personal stories (how people frame their experiences of the ACP process).
- Interpersonal co-creation of accounts (how the researcher’s social identities and positionalities influence the way in which the narratives were co-constructed with participant narratives during data generation).
- Social narratives (how social and cultural contexts influence the ACP process).

Semi-structured, narrative interviews were used to explore PwDs’ and their carers’ experiences of the ACP process to enable a balance between the predesignated question list of the structured interviews and the flexible conversations of the unstructured interviews (Salmons, 2016). This method was also chosen to minimise the likelihood of exacerbating PwD’s symptoms of forgetfulness and anxiety and to accommodate carers busy lives.

As this study took place during the COVID-19 pandemic, when social distancing was mandated in the UK and data protection requirement from the university at the time (2020–2021), online and telephone semi-structured interviews were conducted. Online interviews were undertaken via Microsoft Teams. Telephone interviews were offered as an alternative option to ensure participants who did not want to or could not join online interviews could still participate in the study. A rapid scoping review was undertaken before this study to identify and prepare research protocol that addressed relevant practical, technical and ethical considerations around conducting online interviews and telephone interviews with PwD [23]. Findings from the rapid scoping review were subsequently used to frame the study protocol as well as interview schedules. The interview schedules were tested and revised after four pilot individual interviews with two PwD and two carers.

### Recruitment process

A convenience sampling technique [24] was used to recruit participants via the JDR platform, an online self-registration service endorsed by the Health Research Authority which enables PwD, their carers, friends of PwD as well as healthy volunteers to register their interest in participating in dementia research [25]. PwDs were invited to take part if they had registered on the JDR platform; had been diagnosed with any conditions of dementia; had initiated or discussed an ACP; had access to the internet; lived in Scotland; had a good command of English and were able to give consent.

We asserted that PwD had sufficient capacity to consent at the point of registration on the JDR platform. However, due to their potential fluctuating mental capacity, the primary researcher reassessed PwD’s capacity to consent during the informed consent process with mini-cog instrument [26]. The mini-cog instrument was not used for clinical assessment; rather, it is used as a proxy tool to ensure that PwD could understand the information provided and could recall it. Carers were eligible if they had registered on the JDR platform; were aged over 18; were the primary, unpaid carers of PwD; had access to the internet; lived in Scotland and had a good command of English. In total, 13 PwD were recruited into the study as well as 23 carers.

### Researcher’s social identities that influenced the research process

The primary researcher has both professional and personal experience of dementia and recognised that they could influence the co-constructed narratives with participants. He is a family medicine doctor (general practitioner) and qualitative researcher whose main interest involves dementia, ACP and palliative and end-of-life care. He is also a family member of PwD. As such, he acknowledged that these social identities were influential over the research process before and during the interviews, as well as the data analysis process.

### Data generation process

Interviews with PwD and family carers were arranged in their preferred medium and at their preferred date and time. Prior to the interviews, the primary researcher contacted participants in JDR platform via email and/or telephone. Instructions to join Microsoft Teams meeting were provided, as requested. A reminder email was sent to participants one day before the interview date.

For PwD, those who lived with carers could choose to join the interviews individually or as a dyad. In instances when participants opted for dyad interviews, informed consent was sought from both the PwD and the family carer. For PwD who chose to participate in an individual interview, family carers usually assisted with the informed consent process and prepared the device to join Microsoft Teams. They were not involved during the interview process. This was to safeguard PwD’s confidentiality during the interviews.

During each interview, the support and distressed protocol was utilised throughout. Participants were advised to find a quiet place to join the interview and to use headphones, if possible, to protect their confidentiality. The interviews lasted between 42 and 108 min (mean = 63 min). On conclusion, a verbal debriefing was given and a debrief summary email was sent to participants after each interview. All interviews were stored on OneDrive and subsequently transcribed verbatim. Additional interview field notes were written during and after each interview to aid the analysis.

### Data analysis

Atlas.Ti was used to facilitate the analysis. Transcripts and fieldnotes were uploaded into Atlas.Ti. Each transcript was read line by line; labels were used to identify the speakers. Codes were generated and organised under the three levels of narratives previously described based on Breheny and Wong’s (2018) work.

For the personal stories level of the narrative, the analysis began with the examination of what participants told (the manifest content). Attention was paid to ‘tension’ which was usually the critical moments or the turn of events that were framed by participants which could be the use of repeated phrases (e.g., ‘I had to’), different tones used or silence. These tensions were colour coded for further analysis along with the latent content from the transcripts (what participants implied or omitted) and why the interviews were told in a particular way. For the interpersonal co-creation of accounts level of narrative, the interactions during the interviews when the primary researcher co-constructed the findings with participants that contributed to the research questions were highlighted and analysed. The focus was on how he paraphrased participants’ replies, how certain questions were used to probe their understanding as well as how his positionalities -either as an outsider or insider- influenced the interviews. For the final level of narrative, the social narratives, the focus was on certain interactions from the interviews when participants recounted the sociocultural aspect of their stories and how that influenced the ACP process.

Key persons that were deemed influential to the ACP process and their roles in it were identified. The focus was on how participants interpreted what they perceived as: ACP barriers, ACP facilitators, key persons who influenced the ACP process. This was colour-coded along with how, when and with whom PwD initiated and revised their ACP. Or, when PwD wanted to initiate their ACP and was restricted from others.

The concept of information power was applied to determine the appropriate sample size for the study [27]. The study was anticipated to require a moderate size of participants of more than 10 participants for both PwD and carers based on:

- The narrow study aims.
- Dense sample specificity from participants’ specific experience around ACP.
- The application of Bronfenbrenner’s bioecological theory.
- Medium quality of dialogue from the primary researcher’s background who has both clinical and research experience around ACP, the complex study design and potential fluctuating mental capacity and forgetfulness from PwD.
- Cross case analysis strategy between PwD and carers.

The final number of participants was 28 and was confirmed with subsequent interviews where no new insights were generated.

### Ethical considerations

This study was approved by the University of Dundee School of Health Sciences Ethical Committee (UOD/SREC/RPG/2020/018/Primary researcher’s family name). A flexible informed consent process was implemented. A Participant Information Sheet (PIS) and informed consent form were sent to potential participants one week prior to interview. Participants either provided their written consent via email or they could record their verbal consent immediately before the interview. The primary researcher read and clarified the PIS and informed consent form to potential participants before they agreed to participate. All names are anonymised to protect participants’ confidentiality.

### Participant characteristics

Between October 2020 and March 2021, twenty-one online interviews, ten telephone interviews and one email interview were conducted (see Table 1).

**Table 1:**
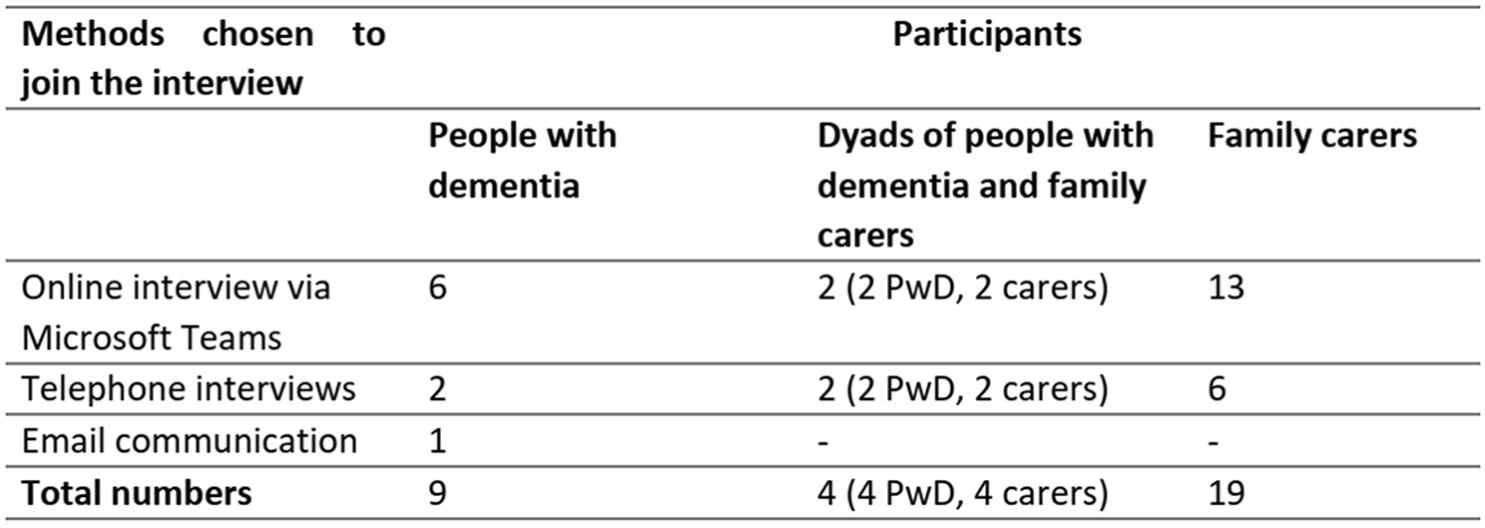
Methods used for interview

Thirteen PwD aged 51 to 87 years (mean = 68.7) joined the study; all were white British. The majority of PwD (*n* = 8) were male. The most common diagnosis was Alzheimer’s disease (*n* = 8). Six PwD were living with young onset dementia. Twenty-three carers aged 41 to 78 years (mean = 59.4) joined the study; all except one were white British. Most carers were female (*n* = 17), were daughters (*n* = 11), or wives/partners (*n* = 6). Participants were distributed across Scotland, ranging from urban areas (*n* = 17), small towns (*n* = 5), rural areas and islands (*n* = 14) (see additional files 1 and 2).

## Results

### Features of the narratives

Direct verbatim quotes from the interviews are used to illustrate the findings and facilitate the discussion. Bold text was used to indicate what participants emphasised. Words added by the researcher for clarifications are enclosed in [] and […] represents omitted text. Three narratives were generated: (i) Shifting expectations; (ii) Relational interdependency and (iii) Trigger points.

### Shifting expectations

This narrative focuses on insights concerning how PwD’s decision-making and expectations toward their future changed over time as the disease progressed. The shifting expectations are essential to understand since they tend to influence PwD’s ACP process throughout. Under this narrative, two types of plans regarding PwD’s future expectations were identified: predefined discussion and agreement (what PwD had previously expressed and wanted) and interim decisions made by carers (what carers deemed best or thought that the PwD would have wanted).

During the early stage of dementia, daily decisions on routines activities and expectations about their potential future life were typically similar to the pre-diagnosis period and were made by PwD themselves since they were able to express and maintain their physical independence and needs (Basic Activities of Daily Living (ADLs)) as well as complex activities to live independently in the community (Instrumental Activities of Daily Living (IADLs) [28]. Such independence was indicated from Mike who had had the Alzheimer’s disease for 6 months:

*‘I get up in the morning*, *have breakfast*, *go out and buy a paper at the paper shop’s just around the corner. Then we might be going for a walk or going up a hill and various other things. But nothing much has changed really*, *so far*, *to be honest’.- Mike (69)*, *living with young onset Alzheimer’s for 6 months.*

Mike positioned himself as an unchanged person. However, he hinted at some lingering uncertainty that his daily life had not changed ‘*so far’*, implying his awareness that this could change. Nevertheless, he preferred to avoid discussing and planning for his future self:

*‘Mike: …I try not to think very much about the far…part of the [my dementia] [pause] [abruptly]. Yeah*, *I just don’t like thinking about that. But****for now***, *you know*, *life’s good*, *and we can have a good time. Interviewer: And just focus on the present. Things you can do and so on? Mike: Yeah.*

His uncertainty avoidance was paralleled with the acknowledgement of his decline associated with dementia when he recounted his direct experiences of caring for his parents, both of whom had dementia. This was narrated along with his professional experience to dementia and how he was aware that ‘*things will get worse*’ in the future:

*Mike: I know that this [dementia] is in my family so…[pause]…it’s quite scary.*

*Interviewer: is it scary because you see…what they had become? At the later stage?*

*Mike: [nodded] Yeah […] And as a Church of Scotland minister*, *I have seen it a lot. I…took …[searching for words]…funeral services for many*, *many people who have been having this sort of (diagnosis of dementia) so yeah… (I know what I will become in the future) […] [I’m aware that] things [my dementia] will get worse. But for the moment*, *I try and live in the moment these days and just not to…get ahead of myself.*

Nevertheless, despite Mike’s personal and professional experiences with PwD, he appeared to avoid planning for his imagined future self and preferred ‘*not to get ahead’* of himself, indicating an ongoing avoidance of the future and focusing on the present.

Mike’s coping mechanism is similar to Jodie who preferred to ‘*take every day as it comes’*:

*I do what I want*, *when I want and it’s great. I don’t think about it [future plans] too much. I just take every day as it come. .- Jodie (56)*, *living with young onset Alzheimer’s for three years.*

Nevertheless, she expressed a declining ability to keep up with her friends due to her dementia symptoms:

*[…] we have our small group [our book club members]*, *sometimes*, *I’m finding it hard to keep up [with conversations] Or…did I miss something*, *sort of feeling. It’s not their fault*, *it’s just them talking as normal (but I still cannot keep up with them).*

Conversations with both Mike and Jodie revealed the story of individuals that continued to take control of their routines despite the lingering uncertainty towards the future or fading independence. At this early stage of dementia, participants’ expectations for the future tended to focus on daily decisions or activities. They preferred not to address future decisions as it required them to envisage a different, perhaps baffling future self, which they preferred to avoid.

As the disease progressed, PwD’s daily decisions would often be made along with and by carers due to PwD’s declining ADLs and IADLs. Carers could act as PwD’s advocate and safeguard the PwD’s decision, similar to Fiona who supported her mother to thoroughly discuss her ACP with the rest of the family:

*She [mum] wants to stay at home for as long as she can. There’s a care home just at the end of the road and she thinks that’s where she should go. She also wanted no resuscitation thing in place. So*, *I’ve just dealt with that. It caused some upset in the family but [the family] do acknowledge it is not their choice – Fiona (59)*, *carer of her mother who is living with Alzheimer’s for 20 years.*

Alternatively, carers might feel that they needed to safeguard PwD, co-create plans with them, or even take over their planning entirely, as indicated by Graeme who decided everything for Beth, his wife who is living with Alzheimer’s disease. Graeme recounted that they did not discuss ACP in-depth at all since Beth felt that she was ‘*not ready’* and *‘not there yet’*. Consequently, all decisions about her daily life as well as decisions about ACP were made and adjusted by Graeme. This stance was grounded in their commitment to each other as spouses which they believed that they were well positioned to make the best decisions for each other:

*The other commitment I made to her [as a spouse] is that “I will not do anything to you or for you that I wouldn’t expect you to do for me if it was the other way around”. So*, *we have that commitment and she knows I wouldn’t (do anything else except that) […] My plan is to keep her home as long as possible*, *with as much help and support as necessary. Until such time*, *if she got to stage that if she was dangerous to herself*, *to me or the neighbours*, *then she may have to go into a more secure environment. But I will keep her at home for as long as possible- Graeme (70)*, *carer of his wife for seven years.*

Graeme’s stance to take over Beth’s decisions contrasted with David who tended to discuss his ACP as a joint changing needs with his wife who is his carer. He recounted how he thoroughly planned his ACP ‘to the last detail’ with his wife as follows:

*David: We [my wife and I] have planned our lives now down to the last detail. Planning is important because you don’t know the route of your journey through dementia whether it’s going to be quick or slow […] My wife says I need to keep a memory box now and this should be useful to her as well as me later on when she wants to remind me of things and I’m keen to do that.[…] We’ve planned. No doubt we’ve made some mistakes but we’re quite proud of the planning that we’ve done. […] We’ve also planned how we’re going to treat each other as life goes on. And she also is not to treat me like a child. If there are any decisions to be made*, *I’d really like to be a part of them.[…] [we planned everything together because] My wife’s opinions are very important*, *they are vital*, *and so is her welfare… –David (66)*, *living with Alzheimer’s for two years*.

David was fully aware of the unpredictable disease trajectory and how his ‘*journey through dementia’* could be ‘*quick or slow’*. Consequently, David used this knowledge as leverage to thoroughly discuss expectations for the future and plan decisions with his wife. Their discussions comprised two types of planning on David’s part: pre-defined discussion (*‘the planning that we’ve done’*) and potential interim and iterative planning for the future (‘*how we’re going to treat each other as life goes on’)*. These different types of planning emphasised the iterative nature of ACPs; that is, ACPs need to be reviewed and, where necessary, revised over time by the persons that would be affected by such decisions. By discussing their plans together, David and his wife could strategise on how to safeguard their decisions about their future lives and, over time, revisit and adjust their plans in ways that are practical for them both whilst accommodating expectations for the future.

Although, their pre-defined discussion may change in the future, these co-created discussions indicate a relational, iterative approach of ACP between David and his wife thus might prove more realistic for both. For example, the extract shows the co-constructed planning process that occurred between David and his wife when she suggested he create a ‘*memory box’*, which he was ‘*keen to do’*. This suggestion highlights the relational nature of the couple; that is, her input also influenced David’s decisions on what he wanted to be enshrined in his ACP as well as potentially affecting both their future lives when ACP decisions may be enacted. As such, David’s future plans did not totally originate from his individual rationalisations, rather, the plans were relationally founded and were originated from his co-existence with his wife (self-in-relation-with others) based perhaps on intimate knowledge of a past lived together.

David’s extract suggests the shifting expectations of his decisions and future plans from individual needs to a shared-decision process with the person who was involved in his care: his wife. This transition juxtaposed and challenged the public narrative of individualistic ACP and emphasised the relational collective shared decision-making between PwD and their key persons. Consequently, this emphasises the importance of PwD’s ACP that will gradually transform over time and need to be co-constructed with others.

### Relational interdependency

This narrative explored the notion of relationality within interrelationships between PwD, their family and HCPs that influence the ACP process. Dementia symptomatology can variably affect how or if a PwD can conceptualise and express their wishes for the future within the context of planning and writing their ACP [8, 29]. Participants suggested several symptoms that can variously influence their ACP over time which are PwD’s declining reading and writing capabilities as well as fluctuating memory. As such, for most PwD, the support from their key person (s) can help to overcome the difficulty surround the ACP process. In this study, the key persons often were PwD’s carers or professionals who have an established, trusting relationship with PwD. Specially, their tacit understanding and appropriate support within the relationship seemed to allow PwD to initiate and revise an ACP accordingly.

One example came from Mary who had difficulty reading writing any documents due to her dementia symptoms but found her dementia link worker useful to support her ACP process:

*Mary: I did them [ACP] with my…dementia worker. Over long periods. And I actually just put it in yesterday.*

*Interviewer: So you and your dementia worker create that together?*

*Mary: Yes we did it together. Well*, *I talked and she typed (laugh) […] ‘cause I struggle (with words and forms) um…that’s another thing. Forms*, *paperwork*, *they’re all of a struggle and so she just asked questions [think]. Yes*, *she asked. We’ve been over questions and then she would…[think] type it.- Mary (51)*, *living with Alzheimer’s for three years*.

From the extract, it appears that Mary’s dementia link worker discussed ACP with her over an extended period, indicating their established relationship. This would have allowed the link worker to understand Mary’s preferences, her ways of communicating and concerns and provide Mary with appropriate support to complete her ACP. This is suggested when the dementia link worker transcribed Mary’s verbal preferences of her ACP (‘*I talked and she typed’*) thus mitigating Mary’s ‘struggle’ to understand words and forms and helping with the ACP documentation process. Therefore, Mary’s ACP was perceived as ‘completed’ due to Mary’s trusting relationship with the link worker and the link worker’s skillsets to support the process.

The interrelationships amongst carers and other family members also influenced the ACP process and how it was likely to be adhered to, as Clara highlighted the ‘*excellent support’* she received from her extended family who adhered to her mother’s ACP which possibly stemmed from her mother’s early discussion of ACP and clear communication to all relevant persons regarding her ACP:

*She [mum] was very clear and communicating to everybody around her*, *how she wanted things to be. As a result*, *they [my aunt and uncles] have been hugely supportive of mum and of me taking care of mum. They have been an excellent support to me. There hasn’t been any conflict or any disagreements about how to proceed with any of her treatment. – Clara (49)*, *carer of her mother for six years.*

Conversely, Donna, who was also caring for her mother. She ‘created’ her mother’s ACP ‘years ago’ with her sister yet her male siblings were not involved nor engaged with the discussion. Consequently, the lack of engagement from her male siblings resulted in a ‘fighting’ in her family over their mother’s care due to ‘different understanding’ of their mother’s conditions and changes that needed to be made:

*I think what was lacking is [us siblings] having some day sitting down and being a little devil’s advocate and saying “Well*, *what happens this and what happens that”. It was just decided that ABC will happen*, *and this is what we’ll do [yet things did not happen that way][…] There was a breakdown within our family unit [after I revised mum’s ACP without telling my brothers]. A lot of fighting*, *which probably could have been avoided if things were just different and we thought things through…-Donna (50)*, *carer of her mother for 15 years*.

These extracts highlighted the complex interrelationships between PwD, carers and extended families that influenced the ACP process. Primary carers might feel the need to initiate an ACP with PwD, but their intentions might not be in line with the extended family. Consequently, the ACP might not be initiated due to this divergence.

Similarly, the extended family might perceive the initial ACP discussion as final. Yet PwD and carers may wish to revise their ACP to make it more reflective of changed circumstances. This intention could potentially be misunderstood by the extended family, thus creating discrepancies in care or disagreement over the ACP.

The perceived variable degrees of support from HCPs can function as either facilitators or inhibitors of the ACP process. Several participants framed their understanding of this phenomena as ‘postcode lottery’ effect. This conceptual understanding differs from the literature which defines the phrase as “*variations in health care between different geographical areas that appear arbitrary and unlinked to health need”* [30] (p.1). In this study, the effects of ‘postcode lottery’ goes beyond the geographical areas and included the relationships between participants and HCPs as well as the perceived variable quality of service that participants felt.

The ‘postcode lottery’ effect was suggested by Mary when she recounted the contrasting experiences of positive support from her dementia link worker and the lack of support when caring for her mother-in-law, who also had Alzheimer’s disease:

*Mary: The support here is very good whereas where I used to stay*, *I wouldn’t have*.

*had what I have here and that’s only…less than 20 miles [away] and the support is just*.

*totally different! I count myself lucky. I’ve got my dementia worker*, *I’ve got a CPN nurse. I have*.

*my consultant […] I don’t know about all these things when I cared for my mother-in-law. I didn’t know about carer support*, *[dementia support] groups and things. I think it’s a postcode lottery*, *to be honest.*

As a PwD, Mary had already completed her own ACP. Yet her experience as a family carer of a PwD was different. Mary did not find the support from HCPs for her and her mother-in-law helpful; Mary was not aware of the support to which she and her mother-in-law were entitled (‘*carer support groups and things’*). As such, this resulted in the lack of ACP discussion between Mary, her mother-in-law and HCPs. She pointed out that the difference in support was based on the area that she lived and how that had a direct impact on her ACP process. This created a surprising contrast in support from HCPs between two areas which were ‘*less than 20 miles away*’ from each other. Hence, Mary framed her situation as ‘lucky’ as it was unclear why she and her mother-in-law received different levels of care and support that PwD are entitled to.

### Trigger points

This narrative encapsulates the timing and reasoning behind the initiation and revision of ACP and examines if an optimal time for the process can be deduced. The majority of participants recounted pivotal moments that triggered the initiation and revision of ACP— the ACP trigger points — throughout the dementia journey ranging from before dementia diagnosis, immediately after the diagnosis or years after. These ACP trigger points encompassed broader discussions beyond medical aspects, emphasising the totality of ACP.

Initially, the contents of ACP that participants discussed tended to be based on broad discussion towards future scenarios and lacked details. Donald who was caring for his wife (Jackie, living with Alzheimer’s disease) created their ACPs together prior to Jackie’s diagnosis after his retirement, making this their first trigger point:

*Once I retired*, *we tried to get some of these [ACP] sorted out so it would be about 2000 and…14-15-ish (2014 or 2015) when we did that? Then we updated it. We transferred it to the local solicitor when we moved [to where we live right now]*, *so that’s been in place about five years. We were prompted by the fact that my parents had left it rather late and it was all a bit difficult towards the end*, *so we determined to be proactive*, *so probably predated Jackie’s dementia’. – Donald (70*, *carer of his wife) and Jackie (68*, *living with Alzheimer’s for five years)*.

During 2014–2015, Jackie had not been diagnosed with dementia yet. As such, their ACP discussion was focused on non-medical aspects of their situation which was adequately reflective of Jackie’s needs. Later, they revised Jackie’s ACP with her GP to include her future care and end-of-life care preferences one year after her diagnosis of dementia (e.g., their second trigger point):

*Jackie: It was the GP [that started the ACP conversation]. She said had I had the plans [ACP]*, *or did I have any thoughts about the future. And we talked about it and she wrote down this (my DNACPR). [speaking to Donald] She wrote down this plan*, *wasn’t she?*

*Donald: Yes*, *she did this [pointed to Jackie’s ACP document] (reading out Jackie’s ACP documents) […] There is a note on the bottom which says*, *“please involve the patient and husband in all health care decisions”. It does include*, *I think*, *a sense of discussing with relatives*, *so that we can…not overrule but*, *but perhaps interpret sensibly*, *what Jackie’s [wishes are] about?*

The couple included Jackie’s future care preferences and explicitly stated in the ACP document that Donald would be involved with her future decisions. As such, Jackie’s revised ACP had changed from the original one in 2014 and was more reflective of their ‘current’ wishes between Donald and Jackie’s, as seen from the note on the bottom of her ACP. After this revision, Donald pointed that he should be able to safeguard Jackie’s preferences as to what she would have wanted (‘*not overrule but*, *but perhaps interpret sensibly*’).

Participants often considered the ‘official diagnosis’ of dementia as another trigger point for ACP discussion. This moment led to either the initiation or revision of ACP. PwD who had already had any prior discussion on their ACP, the discussion afterwards tended be expanded to include place of care and end-of-life care, which, again, emphasises the iterative nature of ACP:

*Clara: At her diagnosis [when mum created her ACP with me]. She was very determined to write her will and deal with power of attorney*. *[…] She was pretty vocal about how she wanted me to support her.*

Apart from the official moment of diagnosis, several PwD interpreted other key poignant situations as their ACP trigger point. Such situations could be years after the diagnosis, as told by Christopher:

*Christopher: [when I realised that I needed my ACP] At the conference. [I met the support group for] some of the…some people with dementia and…few of them [PwD] are dead and put everything in place. And that’s when. it made me realize it [death] could…err…happen at any time. You never know your minutes. So…get it [ACP] in place now because it’s going to save a lot of heartaches…and confusion…and stuff […]*.

*Interviewer: was it your wife that you discussed your plan with?*.

*Christopher: Me wife and…[think] meself*, *me son and daughter. We sat down and discussed… [searching for words] it with them.[…] It’s already been in place [ACP]. I’ve got one for me health*, *and I’ve got the errr do not resuscitate.[DNACPR] in place. We did that about after a few years [after my diagnosis]. –Christopher (68)*, *living with Alzheimer’s for 11 years*.

Christopher retold the key moment of realisation happened to him at a conference years after his diagnosis when the death of other PwD were the main trigger that enabled him to become aware of the benefits of ACP (‘*put everything in place’*). As such, to ‘*prevent the heartaches and confusion*’ within his family, he decided to discuss ACP together with others.

**Fig. 2:**
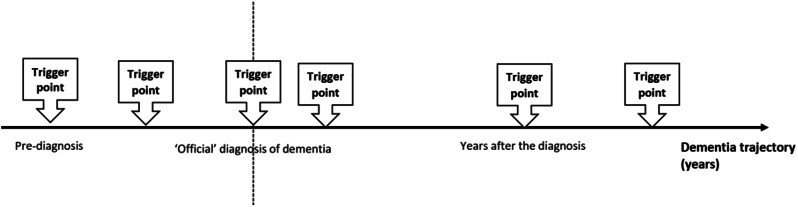
The ACP trigger points throughout the dementia trajectory

**Fig. 3:**
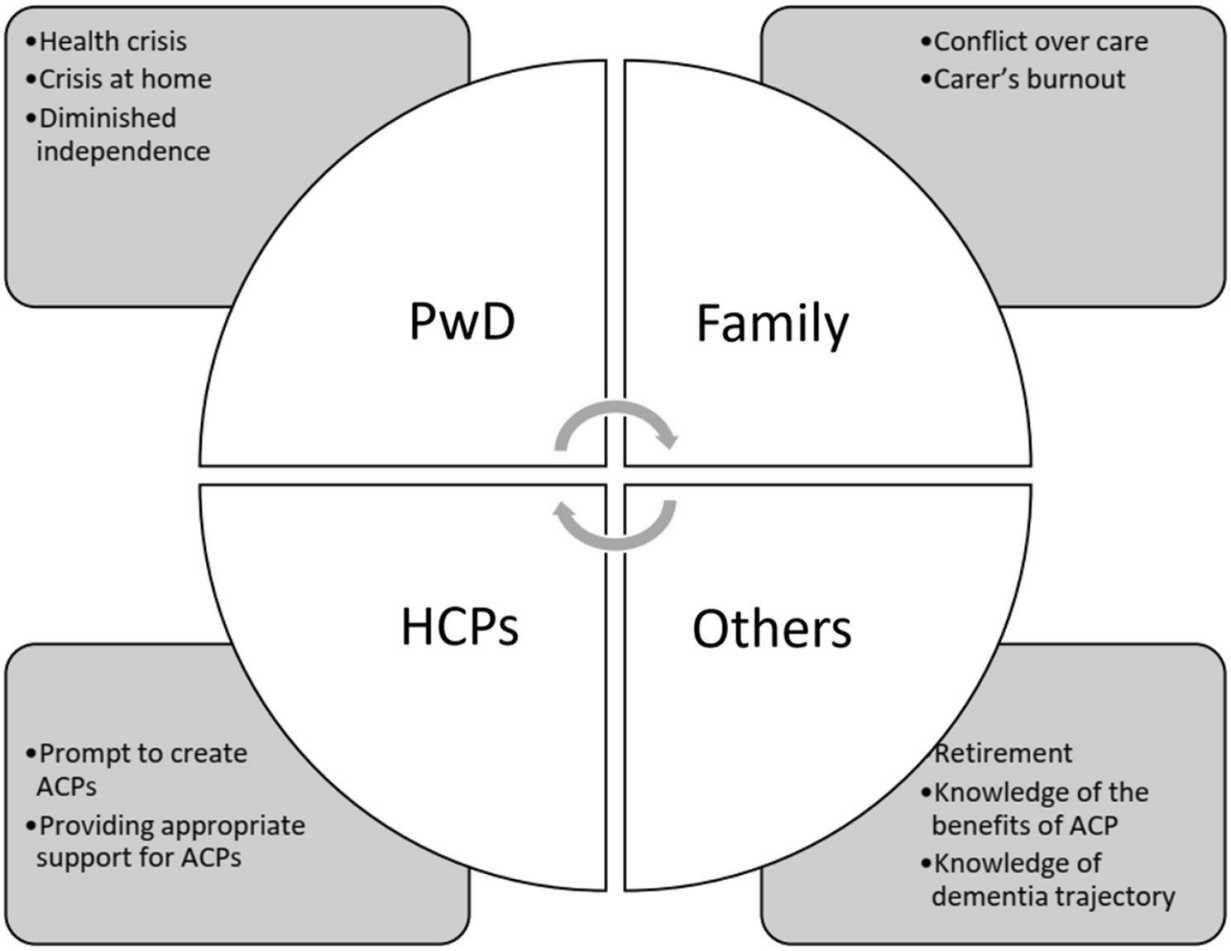
Reasons and situations around the ACP trigger points

These extracts suggest various events that acted as ACP trigger points that triggered PwD to conceptualise, initiate or revise their ACP across the disease trajectory. As such, it is highly unlikely to locate a generic, single ideal ‘window of opportunity’ period for PwD to discuss their ACP. The ACP trigger points from this study are summarised in Figs. 2 and 3, illustrating when participants had discussed their ACP throughout dementia trajectory and the situations around their trigger points (see Figs. 2 and 3).

## Discussion

This section respectively discusses each research question. For the first research question, ‘How do PwD’s daily lives change over time and how do these changes affect their expectations for the future?’, the narrative of shifting expectations suggested PwD’s shifting expectations of their future across the disease trajectory. Initially, PwD who were at the early stage of the disease could maintain their daily activities and routines prior to the diagnosis. As such, their expectations for the future were based on a continuation of their pre-diagnosis period. They did not feel the need to visualise the projection of their future self since doing so meant that they had to conceptualise activities and identities that would be beyond their established routines and current normality. This process coheres with Han et al.’s (2015) description of ‘*engagement for continuity’*; that is, PwD preferred to engage with activities that enabled them to feel connected with themselves, others and familiar environments [31]. This engagement facilitated the maintaining of pre-diagnosis identities and meaningful relationships with families and communities. Consequently, the lingering uncertainty towards decisions for the future remained throughout but had not yet been explicitly engaged.

As the symptoms of dementia began to affect PwD’s routines and interim decisions in their daily lives, their decisions and expectations appeared to shift from their pre-diagnosis individual decisions and expectations into relational decisions that were co-created with others. Both their pre-defined discussion and agreement, interim decisions and future expectations were often negotiated with key persons, who were usually carers due to their relationships and interdependence [32, 33]. This shift is relational; that is, the extent of PwD’s individual pre-defined discussion and agreement would decrease when carers had to increase their support as the disease progressed. As such, any decisions that were made by PwD would, inevitably, involve other stakeholders; in this case, carers. At this point, the ACP process could shift into shared-decision making between PwD and carers. This has been proven as a practical ACP discussion model for PwD and carers, given that they can collaborate and co-create ACP and expectations for the future together [34]. An ACP that was created and discussed this way would ensure that carers could safeguard or adjust any expectations in the future when PwD became more advanced [35].

During this shift from individualistic ACP to relational ACP, the decisions that were previously discussed and negotiated would be likely to be adhered to if both PwD and their families had extensively planned ACP together and made the plans explicit. The findings resonated with the literature where extensive ACP documentation and revision has been found to be conducive to ACP concordance [34, 35]. As such, extensive ACP discussion and documentation revision may be necessary to ensure that PwD’s preferences are practical and relational to what families can accommodate.

For the second research question,’ How does the social context of PwD affect the initiation and revision of their ACPs?’, the relational interdependency narrative frames the variable interrelationships between PwD, families and HCPs that influenced the initiation and revision of their ACPs. As seen from Mary’s account, for PwD, their initiation and revision of ACP tend to be affected by the variable decline from their dementia symptoms [29]. Yet, this decline seems to be mitigated by their key persons who could provide appropriate, relational support for PwD to initiate and revise their ACP. As such, the trusting relationship with and support from PwD’s key persons seemed to be crucial to ensure that PwD had sufficient support for their ACP process [33, 36]. Findings seem to suggest the relationality of PwD’s ACP and how such ACP is embedded within several interrelationships and interdependency.

This interdependency was further explored when the ‘postcode lottery’ effect also had an influence over the ACP process for PwD; that is, PwD who had efficient support from HCPs tended to have a higher chance to discuss their ACPs. One explanation might stem from the way in which HCPs interpreted and differently implemented the policies in their practice [37] thus resulting in the variable ACP support for PwD. However, the perspectives of PwD and families from this study could not fully explain this arbitrary effect on the ACP ‘postcode lottery’ effect thus requires further examination.

The issue of temporality around ACP was central to the last research question: are there optimal times for initiating and revising ACPs with and for PwD?. This was answered via the identification of ACP trigger points which were indicated throughout the disease trajectory hence challenges the recommendations from the past literature that overly emphasised the early initiation of ACP [29, 38, 39]. That is, PwD can successfully discuss their ACP years after their diagnosis as long as appropriate relational support is provided. As such, the identification of trigger points may prove useful as an alternative strategy to discuss an ACP with and for PwD i.e., during PwD’s health crisis, diminished independence or family conflicts over PwD’s care [36, 40].

Another finding that offers insight regarding the timing to revise an ACP can be derived from the particular ways PwD define the term ‘ACP’ since participants framed the meaning of ACP differently from the officially recognised definition used by HCPs. To participants, the meaning of their ACP might only cover the notion of appointing a power of attorney guardian or creating a will. However, the analysis indicates that their understanding of ACPs can change over time and additional content may then be considered. This finding mirrors the concept of ACP that goes beyond the preferences of medical care towards end-of-life [33, 38]. Yet, in practice, HCPs tend to ignore this comprehensive perspective of ACP and focus on medical aspects such as treatment, place of care or end-of-life care preferences. As such, this can potentially create mismatched expectations between the PwD, their family and HCPs. Given that different aspects of ACP were perceived as essential and relevant to different stakeholders that potentially lead to unfulfilled needs of PwD that do not fully address their personal values, life goals and preferences over future care.

### Strengths and limitations

This theory-informed study utilised Bronfenbrenner’s bioecological systems and offered more robust findings and suggestions i.e., the impact of the ‘postcode lottery’ effect that influenced the ACP process for PwD was indicated under the exosystem and macrosystem. Moreover, two triangulation techniques - methodological triangulation and data triangulation - were undertaken which increased the robustness of the findings [41]. Methodological triangulation was achieved via the use of online interview and telephone interview methods. The data triangulation was utilised from several data sets: (i) interviews transcripts of PwD (ii) interviews transcripts of carers and (iii) interviews transcripts of dyads of PwD and carers.

Next, the use of the narrative allowed for co-creation of the complex conceptual understandings around ACP with both PwD and carers’ input. This is beneficial because the ACP process amongst PwD inevitably involves input from their family as the disease progresses [33]. The poignant, critical moments over the disease progression that acted as the ACP trigger points for participants were also captured with this approach.

The online method also enabled the participation of PwD who are often be hard-to-reach due to family or professional gatekeepers [43–45] thus making the study more inclusive. This decision was practical, ethical and legally compliance for both participants and the research team since the interviews took place during the lockdown restrictions across the UK (October 2020- March 2021) when the UK government had not fully implemented the COVID-19 vaccination rollout programme yet.

Nevertheless, this study poses several limitations:

First, participants were ethnically homogenous; all except one participant were white British. Consequently, the findings were unable to fully capture the cultural nuances and family dynamics from other non-white populations that might influence the initiation and revision of ACP. This is important to address in future studies given that UK populations are more diverse and do not consist of White British only and research from people from the ethnic minority background is still underrepresented [45]. Second, relatively fewer numbers of PwD joined the study. This limitation was mitigated with the application of information power and the narrative approach. Third, poor internet connection resulted in lost data from the interviews. This is one of the inherent limitations of online interviews [46, 47] and was mitigated when the primary researcher asked participants to repeat some parts of the interviews again. Finally, participants were recruited via JDR platform thus excluding some potential participants who were not registered in the platform. To mitigate this limitation in future studies, a more inclusive recruitment strategy is needed such as participant recruitment outside the JDR platform and via other dementia networks.

### Future implications

There are several participants (6) who has been living with young onset dementia. Given that people who are living with young onset dementia have different disease trajectories and needs, compared to older PwD [33, 48], their ACP are likely to differ from PwD who is more than 65 years of age. Therefore, the nuances around people with young onset dementia’s understanding regarding the initiation and revision of ACP should be further explored. Next, the study did not include HCPs or policymakers. Future studies could explore their perspectives for a more comprehensive understanding around the ACP process for PwD.

Moreover, the gender of the participants was reasonably well-distributed (14 male, 22 female). This is potentially beneficial and could provide additional insight to dementia research landscape given that PwD tend to be female yet the research and policies seem to present and analyse PwD as gender neutral [49]. This gender-neutral approach to dementia might miss the impact of gender regarding dementia care. However, we did not design nor intend to analyse the data with a gendered lens. Consequently, this approach to analysis -via the lens of gender - could potentially yield additional insights in future research. Finally, a longitudinal study design may offer additional insights around the ACP trigger points further.

## Conclusions

This study highlighted the changing co-constructed needs as the disease progresses between PwD and their families which influenced how PwD initiate and revise their ACP. The identification of ACP trigger points - the pivotal events throughout the dementia journey - along with the timing and reasoning that prompted PwD and family members to initiate or revise their ACPs were also discussed and examined. Such trigger points indicated that PwD can initiate and revise their ACPs throughout the disease trajectory provided relational support is available and key persons involved in their care are involved and agree with the decisions being made. Consequently, an alternative, relational approach to ACP with and for PwD is recommended. This approach positioned HCPs who have established, trusting relationships with PwD and carers to provide relational support for PwD to support their symptoms and should enable PwD to initiate and revise an ACP with their family whilst ensuring that their life goals are part of engagement and discussion during the process.

## Electronic supplementary material

Below is the link to the electronic supplementary material.

Supplementary Material 1: Additional files: Dementia participants’ characteristics. Family carer participants’ characteristics. Interview schedule for PwD. Interview schedule for carers.

## Data Availability

The data generated and analyzed during the current study are not available for public use, due to confidentiality, but are available from the corresponding author, TP, on reasonable request.

## Abbreviations

ACP: Advance Care Planning
ADL: Activities of Daily Living
IADL: Instrumental Activities of Daily Living
HCPs: Healthcare professionals
JDR: Join Dementia Research
PwD: People living with dementia
UK: United Kingdom
