# Supplementary material for: A relational approach to co-create Advance Care Planning with and for people living with dementia: a narrative study
Source: BMC Palliat Care. 2025 Jan 8;24:5. doi: 10.1186/s12904-024-01632-y (PMC11708118; doi:10.1186/s12904-024-01632-y)

Additional file 1. Dementia participants’ characteristics


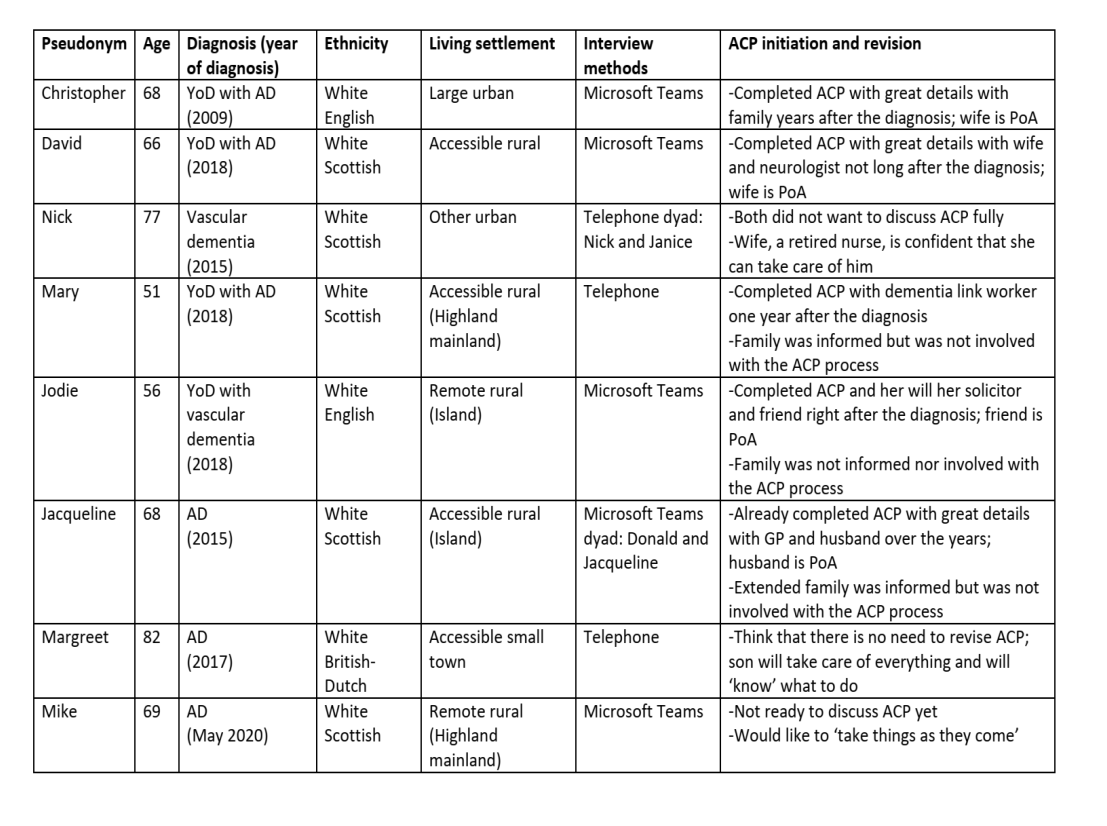


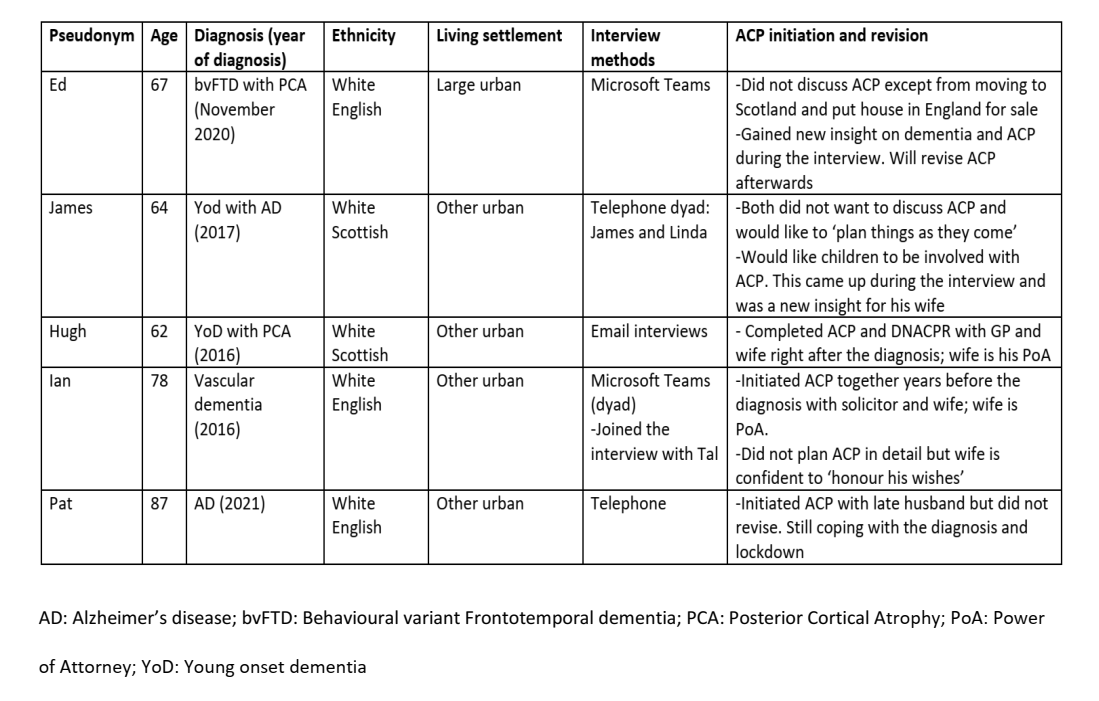


Additional file 2. Family carer participants’ characteristics


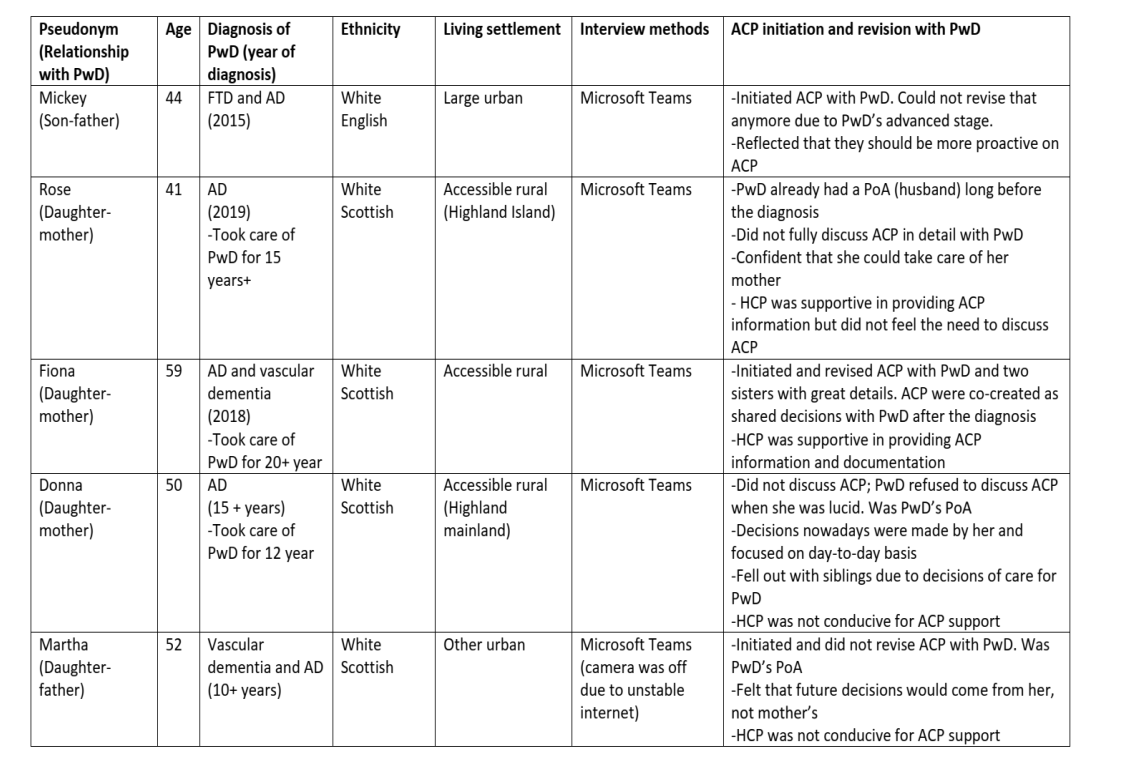


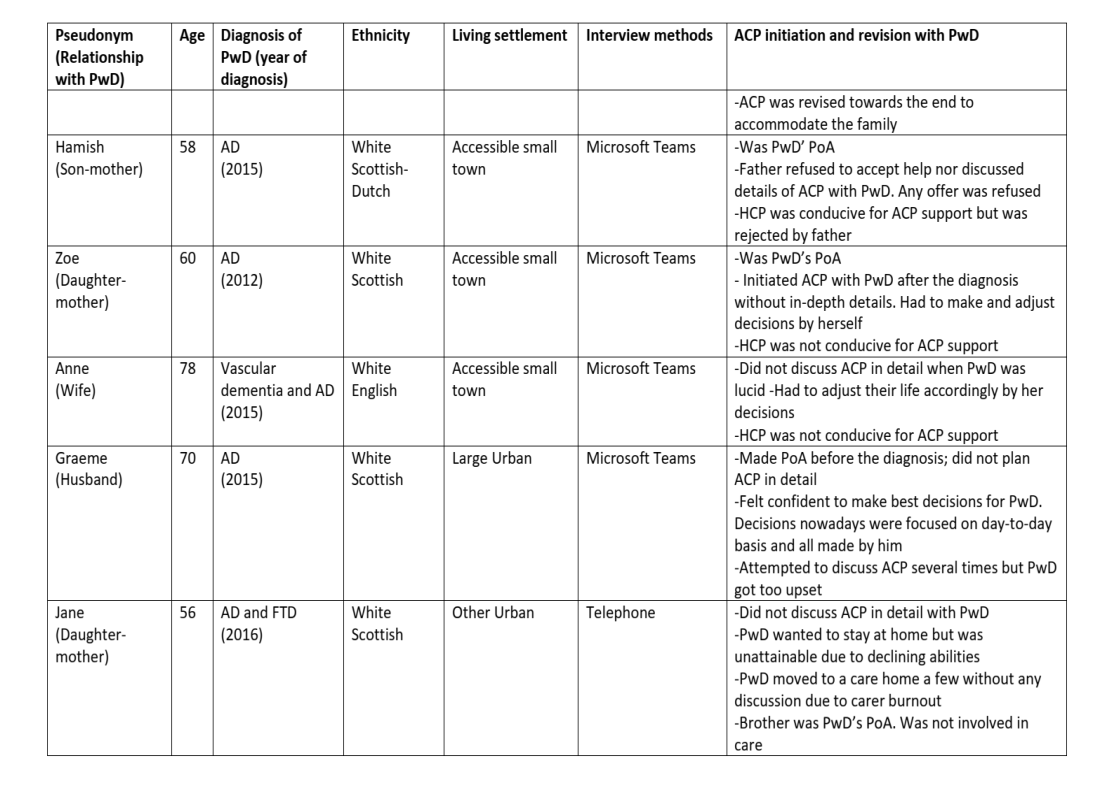

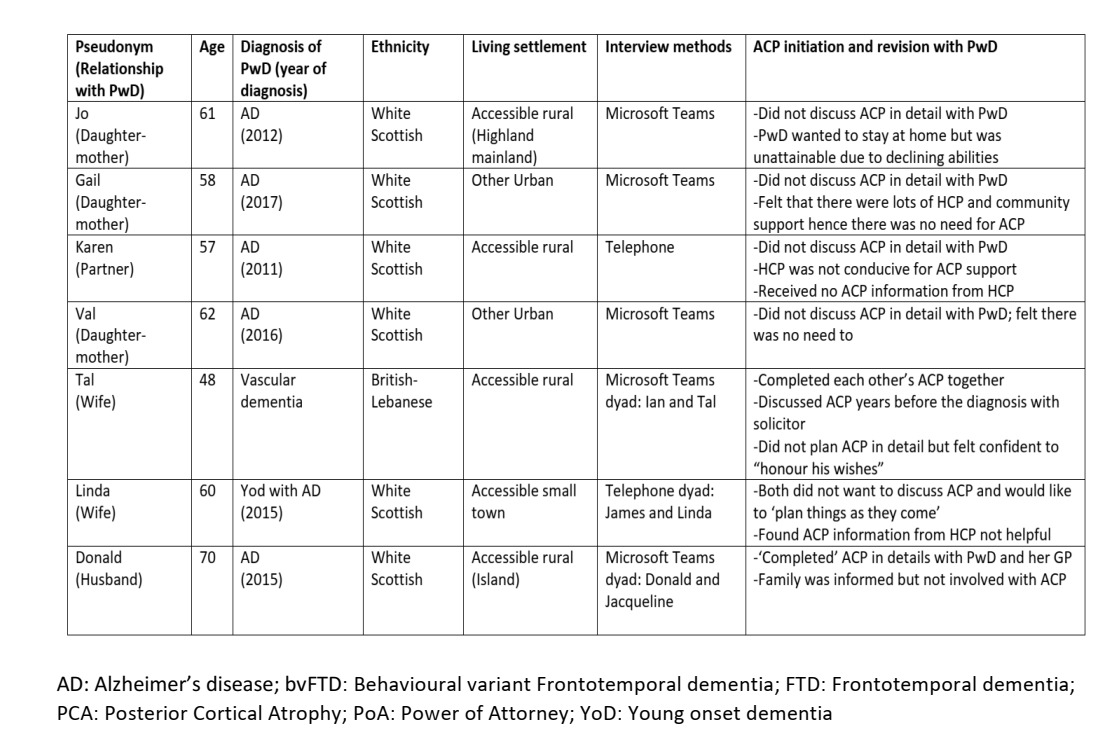

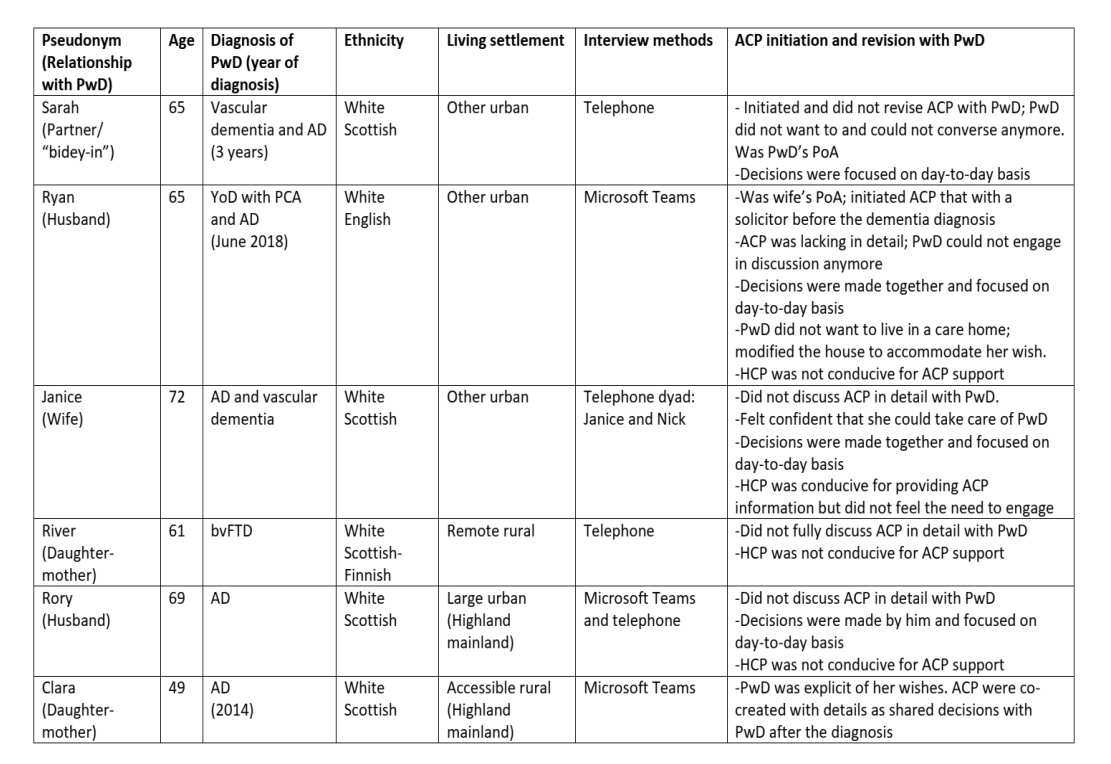


Additional file 3. Interview guide for people living with dementia


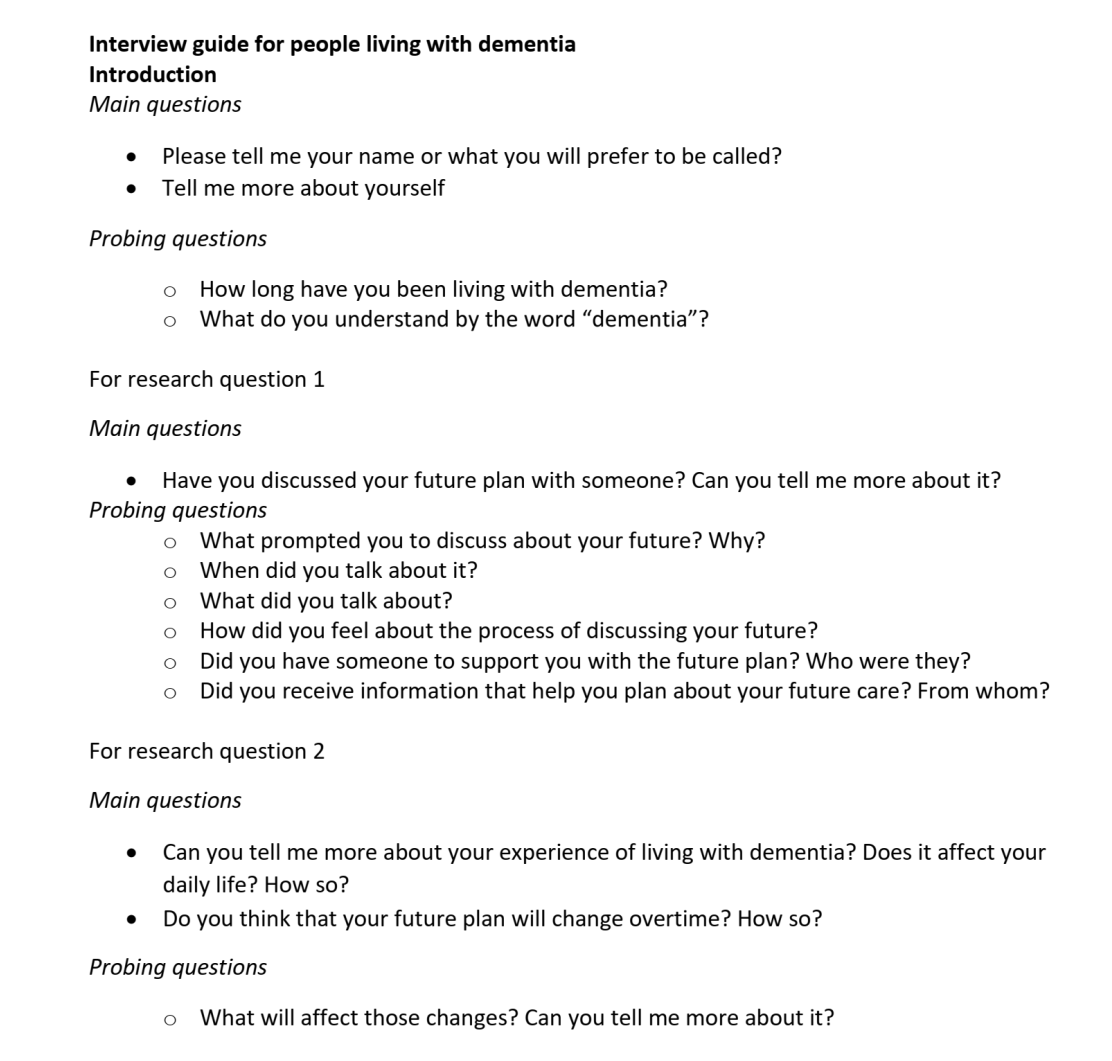


Additional file 4. Interview guide for family carers


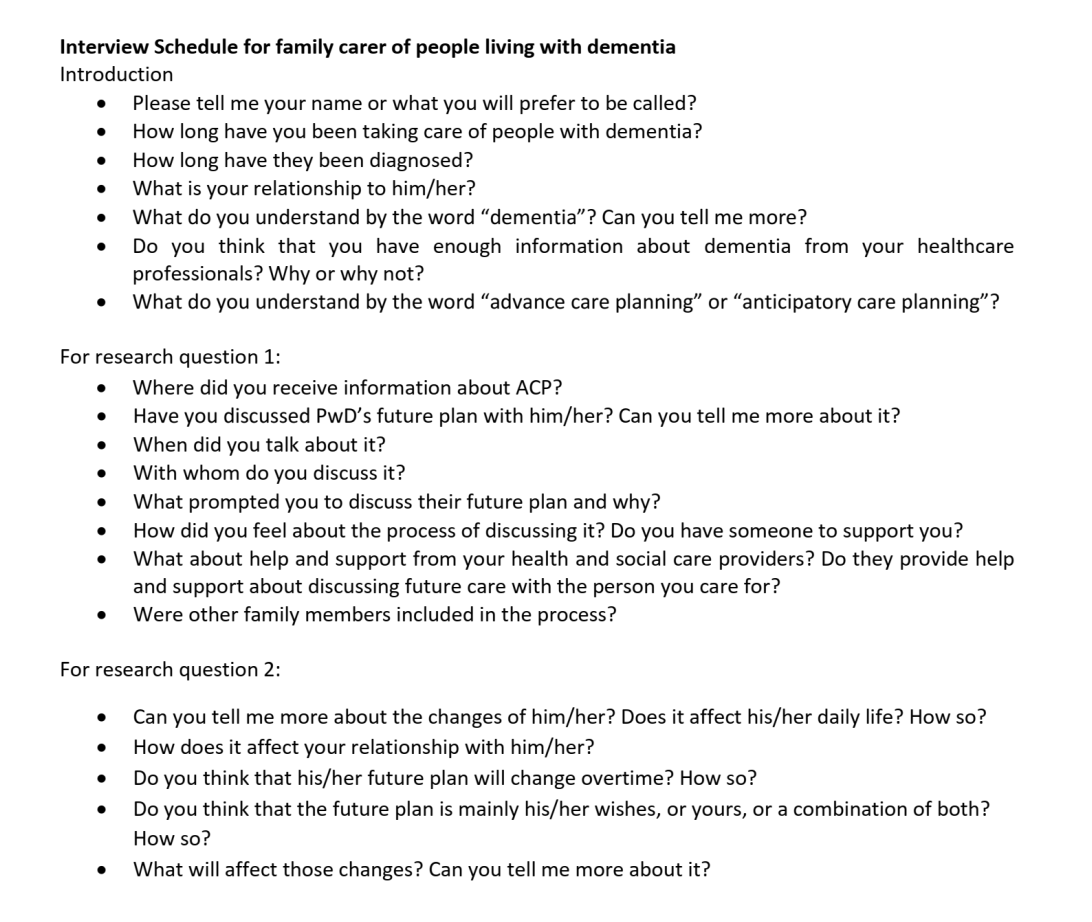


Additional file 5. Debrief form


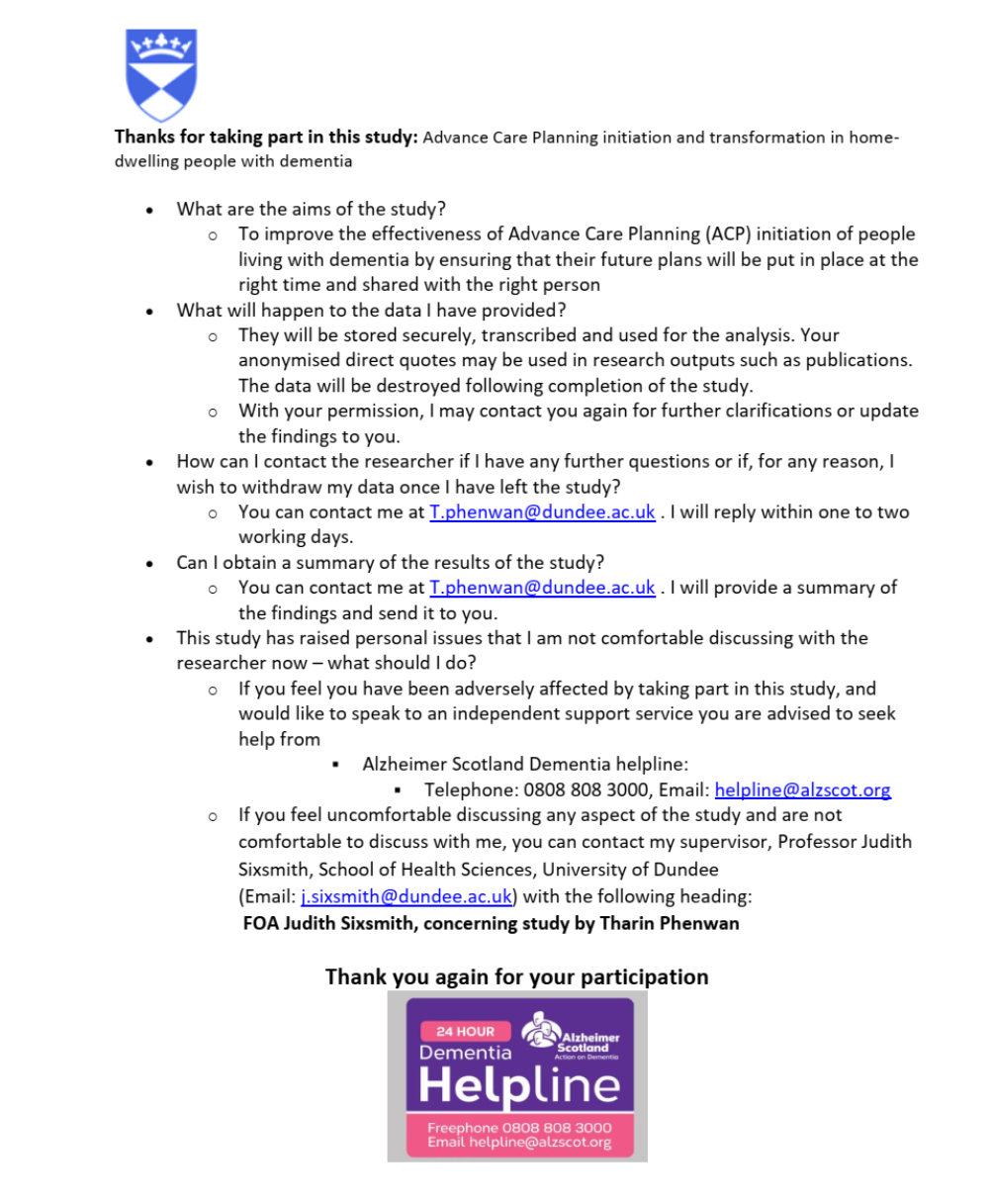

Supplement: Supplementary file 1 — Supplementary Material 1: Additional files: Dementia participants’ characteristics. Family carer participants’ characteristics. Interview schedule for PwD. Interview schedule for carers. [file 12904_2024_1632_MOESM1_ESM.docx]
